# The JAK/STAT Signaling Pathway Mediates Antibacterial Immunity in the Soybean Aphid *Aphis glycines*

**DOI:** 10.3390/insects17070687

**Published:** 2026-07-01

**Authors:** Zhengbing Wang, Xin Miao, Zhen Li, Jiahui Zhang, Manman Zheng, Wenkai Bu, Lei Yang, Kedong Xu, Xiaoyue Sang, Keshi Ma, Mingsheng Yang

**Affiliations:** 1 College of Life Science and Agronomy, Zhoukou Normal University, Zhoukou 466001, China; wangzb@zknu.edu.cn (Z.W.); 15515675276@163.com (X.M.); lizhen920110@126.com (Z.L.); zhangjiahui0806@163.com (J.Z.); 20191068@zknu.edu.cn (M.Z.); 18737262783@163.com (W.B.); 17518956270@163.com (L.Y.); 2 Field Observation and Research Station of Green Agriculture in Dancheng County, Zhoukou Normal University, Zhoukou 466001, China; 3 Key Laboratory of Plant Genetics and Molecular Breeding, Zhoukou Normal University, Zhoukou 466001, China; xukd1107@126.com; 4 Henan Key Laboratory of Crop Molecular Breeding and Bioreactor, Zhoukou Normal University, Zhoukou 466001, China; 5 College of Journalism and Communication, Zhoukou Normal University, Zhoukou 466001, China; sangxy521@163.com

**Keywords:** *Aphis glycines*, JAK/STAT pathway, immune signaling, antibacterial defense, RNAi, biological control

## Abstract

The JAK/STAT pathway is a conserved signaling pathway involved in insect immune regulation. Although its antibacterial role has been investigated in some aphids, evidence is lacking for *Aphis glycines*. In this study, we identified JAK/STAT pathway-related genes in *A. glycines* and examined their roles in antibacterial defense. Candidate genes were identified and characterized through sequence, domain, and phylogenetic analyses. Developmental expression and transcriptional responses to bacterial infection were determined by qRT-PCR, and gene function was evaluated by RNA interference followed by bacterial challenge. Six putative JAK/STAT pathway-related genes were identified, including *AglyJak*, *AglyDome-1*, *AglyDome-2*, *AglyDome-3*, *AglyStat92E-1*, and *AglyStat92E-2*. Conserved domain architecture and phylogenetic analysis confirmed these genes as *A. glycines* JAK/STAT orthologs. Expression profiling showed that, except for *AglyJak* and *AglyDome-2*, most genes were most highly expressed in early nymphal stages, particularly in first-instar nymphs. After injection with *Escherichia coli* or *Staphylococcus aureus*, the transcript levels of *AglyJak*, *AglyDome*, and *AglyStat* genes increased significantly. Gene silencing significantly reduced aphid survival following bacterial infection. These results indicate that JAK/STAT pathway-related genes participate in the antibacterial defense of *A. glycines* and provide candidate targets for further studies on immune regulation and biologically based management of this pest.

## Simple Summary

The soybean aphid is an important pest of soybean, and its control still relies largely on chemical insecticides, which can lead to resistance and environmental concerns. A better understanding of the immune system of this insect may help identify new targets for sustainable pest management. In this study, we identified six genes related to the Janus kinase/signal transducer and activator of transcription signaling pathway, an immune communication system that helps insects respond to infection. Most of these genes were highly expressed in young aphids and were strongly activated after infection with two common bacteria, *Escherichia coli* and *Staphylococcus aureus*. When these genes were reduced using a gene-silencing method, aphid survival after bacterial infection decreased significantly. These results show that this pathway contributes to antibacterial defense in soybean aphids and may provide useful targets for future biologically based control strategies.

## 1. Introduction

The soybean aphid, *Aphis glycines* Matsumura (Hemiptera: Aphididae), is among the major pests of soybean (*Glycine max*) in North America and East Asia [1,2]. It feeds on the sap of leaves, stems, and pods, resulting in leaf curling, reduced pod set, and yield losses [3]. In addition, the soybean aphids can transmit plant viruses [4]. Currently, the control of *A. glycines* mainly relies on chemical insecticides as the key measure of integrated pest management (IPM) [3,5]. However, the long-term use and improper application of insecticides can promote the development of resistance, thereby reducing control efficacy [5,6,7]. Therefore, exploring key molecular targets closely related to the survival and adaptation of the soybean aphids and developing more selective and sustainable control strategies have become important research directions.

To identify such molecular targets, it is essential to understand the biological systems that support aphid survival under environmental and microbial challenges. Unlike vertebrates, insects lack a traditional adaptive immune system and rely instead on innate immunity to resist pathogen infections [8,9]. The innate immune system comprises cellular responses, such as phagocytosis, encapsulation, and nodule formation, as well as humoral defense processes, including melanization, reactive oxygen regulation and the induction of immune-related genes [10,11]. Insect innate immunity is broadly divided into cellular and humoral responses. The regulation of humoral immunity in insects depends on multiple signaling pathways. Examples include the Toll, immune deficiency (IMD), and Janus kinase/signal transducer and activator of transcription (JAK/STAT) pathways [11,12]. Activation of these pathways enhances innate immune responses in insects [13]. Among them, the JAK/STAT pathway is a relatively conserved signaling pathway in insects [14]. In model insects, the Toll, IMD and JAK/STAT pathways together constitute the core framework of the immune regulatory network [15]. The JAK/STAT pathway is involved not only in host defense but also in developmental regulation, stress responses, and tissue homeostasis [16,17]. Therefore, this pathway represents an important molecular link between insect immune responses and physiological adaptation.

Given the central role of JAK/STAT signaling in insect immunity, studies across diverse insect species provide an important framework for understanding its potential functions in the soybean aphids. Previous research has shown that the JAK/STAT pathway is broadly involved in the responses of insects to various pathogens. In *Drosophila melanogaster*, cytokine-like unpaired ligands activate the Domeless receptor, leading to the recruitment of JAK, also known as Hopscotch, and the transcription factor Stat92E, ultimately inducing downstream immune genes [18]. In mosquitoes, activation of the JAK/STAT pathway enhances antiviral resistance, whereas inhibition of this pathway increases susceptibility to viral infection [19]. Similarly, in lepidopteran insects, the JAK/STAT pathway responds to bacterial and fungal stimuli and participates in postinfection immune regulation [20,21]. Collectively, these studies demonstrate that the JAK/STAT signaling pathway plays a conserved and crucial role in mediating the immune responses of insects to diverse pathogens, including bacteria, fungi, and viruses.

Although these studies highlight the conserved role of JAK/STAT signaling in insect immunity, aphids appear to possess several distinctive immune characteristics that make this pathway especially worth examining in this group. Compared with holometabolous insects, aphids have a reduced repertoire of classical antimicrobial peptides and lack certain components of the IMD pathway, suggesting that their antibacterial defense may depend more heavily on other signaling pathways and alternative regulatory mechanisms [22,23]. Studies of the pea aphids (*Acyrthosiphon pisum*) and the peach aphids (*Myzus persicae*) have revealed that JAK/STAT pathway-related genes can be induced by *Escherichia coli* and *Staphylococcus aureus* [24,25]. Furthermore, silencing these genes significantly reduces aphid survival under bacterial challenge [24,25]. These findings support an important role for JAK/STAT signaling in aphid antibacterial defense.

Despite these advances in other aphid species, the immune signaling mechanisms of the soybean aphid remain poorly understood. Although the agricultural importance of the soybean aphids has been widely recognized [2], the molecular basis of their immune signaling pathways, particularly the core antimicrobial signaling network, has not yet been fully elucidated. Most studies on this pest focus on ecology, field management, resistance evolution, and RNAi delivery technology [5,26]. RNA interference has emerged as an important approach for precision pest control because of its high sequence specificity [27]. In addition, the development of nanodelivery and transcutaneous delivery systems has provided new platforms for dsRNA application in piercing-sucking pests [28,29]. Given the conserved role of JAK/STAT signaling in immunity, characterizing this pathway in *A. glycines* is a necessary step toward evaluating its potential as an RNAi target. Therefore, identifying and functionally validating antimicrobial defense-related signaling genes in the soybean aphids will not only improve our understanding of the soybean aphid immune physiology but may also provide candidate molecular targets for novel control strategies.

To address this knowledge gap, the objective of the present study was to identify and functionally characterize JAK/STAT pathway-related genes in *A. glycines* and to evaluate their roles in antibacterial defense. To achieve this objective, we identified six JAK/STAT pathway-associated genes in *A. glycines*, namely *AglyJak*, *AglyDome-1*, *AglyDome-2*, *AglyDome-3*, *AglyStat92E-1*, and *AglyStat92E-2*, and analyzed their conserved structural features, developmental expression patterns, and transcriptional responses to *E. coli* and *S. aureus* challenge. In addition, RNA interference followed by bacterial infection assays was used to further assess the contribution of these genes to antibacterial defense in the soybean aphid. Our results showed that these genes are conserved components of the JAK/STAT pathway, respond to bacterial infection, and contribute to aphid survival under bacterial challenge. These findings clarify the role of JAK/STAT signaling in the immune defense of *A. glycines* and provide a foundation for future studies on RNAi-based or biological control strategies.

## 2. Materials and Methods

### 2.1. Insect Rearing

The *A. glycines* laboratory colony used in this study was originally collected from a soybean field in Heihe, Heilongjiang Province, China, in 2020. Aphids were kept on soybean plants in a controlled environment with a temperature of 25 ± 1 °C, 65–70% relative humidity, and a 16:8 h light:dark cycle. For each developmental stage of *A. glycines*, we collected samples from different life stages: first-, second-, and third-instar nymphs (60 individuals each), fourth-instar nymphs (30 individuals), and apterous and alate newly emerged adults (30 individuals each). Each stage was represented by three independent biological replicates. After collection, all specimens were immediately flash-frozen in liquid nitrogen and stored at −80 °C until RNA extraction.

### 2.2. Identification and Bioinformatic Analyses of JAK/STAT Pathway Genes

To identify the core JAK/STAT genes in *A. glycines*, previously reported JAK/STAT-related amino acid sequences from *A. pisum* and *M. persicae* were used as tBlastn queries [24,25]. The soybean aphid genome assembly (GenBank accession no. GCA_009761285.1) was used as the reference database for homology searches, with an E-value cutoff of 1 × 10^−5^. The physicochemical properties of the proteins were computed using the ExPASy ProtParam tool (https://web.expasy.org/protparam/, accessed 12 October 2025). Conserved domains were analyzed using the SMART tool (https://smart.embl.de/, accessed 15 October 2025) [30]. Phylogenetic relationships were inferred from amino acid sequences using the maximum-likelihood method in IQ-TREE v2.4.0 with 1000 bootstrap replicates [31]. The optimal substitution model is provided in Table S1.

### 2.3. Bacterial Challenge

*E. coli* (ATCC 25922) and *S. aureus* (ATCC 25923) were cultured in liquid LB medium at 37 °C with shaking. Cells were harvested by centrifugation during the exponential growth phase (OD600 ≈ 0.8) and resuspended in sterile 0.85% (*w*/*v*) NaCl. The final bacterial titers were adjusted to 1 × 10^8^ CFU/mL for *E. coli* and 1 × 10^9^ CFU/mL for *S. aureus*. Newly molted fourth-instar apterous nymphs were anesthetized with CO_2_ and placed on 2% agar plates. Each nymph was injected in the abdominal tergum with 20 nL of bacterial suspension using an R480 microinjector (RWD Life Science, Shenzhen, China). Aphids injected with an equal volume of sterile 0.85% (*w*/*v*) NaCl served as injection controls. For each treatment, 50 nymphs were used. After injection, the insects were maintained on fresh soybean seedlings under standard rearing conditions as described in Section 2.1. At 6, 12, and 24 h postinjection, 20 surviving nymphs were randomly collected from each group for qPCR analysis. Three independent biological replicates were performed for each treatment at each time point.

### 2.4. RNA Isolation and cDNA Synthesis

Total RNA was extracted from the samples using an Ultra-Pure Total RNA Extraction Kit (Simgen, Hangzhou, China) in accordance with the manufacturer’s instructions. The integrity of the RNA was evaluated by 1.5% agarose gel electrophoresis. RNA concentration and purity were determined using a NanoDrop 2000 spectrophotometer (Thermo Fisher Scientific, Waltham, MA, USA). For each sample, approximately 5 µg of total RNA was reverse transcribed using Hifair^®^ III 1st Strand cDNA Synthesis SuperMix for qPCR (Yeasen, Shanghai, China).

### 2.5. Quantitative Real-Time PCR (qRT–PCR)

Gene expression levels were quantified by quantitative real-time PCR (qRT–PCR) using a CFX96 Touch™ Real-Time PCR System (Bio-Rad, Hercules, CA, USA). Each 20 μL reaction mixture contained 10 μL of Hieff^®^ qPCR SYBR^®^ Green Master Mix (Yeasen, Shanghai, China), 1 μL of cDNA template, 0.8 μL each of forward and reverse primers (10 μM), and 7.4 μL of nuclease-free water. The primers listed in Table 1 were designed using the Primer3 online program (http://primer3.ut.ee/, accessed 18 October 2025). On the basis of a previous validation study in *A. glycines* [32], β-Tubulin (*β-Tub*), Elongation factor-1α (*EF1α*), and 40S ribosomal protein S12 (*RPS12*) were selected as reference genes. *EF1α* and *RPS12* were used for normalization in developmental stage comparisons, whereas *β-Tub* and *EF1α* were used for normalization following bacterial challenge and dsRNA treatment. The thermal cycling conditions for PCR were 95 °C for 3 min, followed by 40 cycles of 95 °C for 10 s, 60 °C for 20 s, and 72 °C for 20 s. Amplification specificity was confirmed by melting curve analysis from 60 to 95 °C with increments of 0.5 °C every 5 s. All reactions were performed in triplicate. Relative expression levels were calculated using the 2^−ΔΔCt^ method [33].

### 2.6. dsRNA Synthesis and Functional Analysis After RNAi

Double-stranded RNA (dsRNA) was synthesized using a T7 High Yield RNA Synthesis Kit (Yeasen, Shanghai, China) with gene-specific primers containing T7 promoter sequences (Table 1). The green fluorescent protein (GFP) gene from the jellyfish *Aequorea victoria* was used as the control template. Synthesized dsRNA was purified, and its integrity was assessed by 1.5% agarose gel electrophoresis; concentration and purity were determined using a NanoDrop 2000 spectrophotometer (Thermo Fisher Scientific, Waltham, MA, USA).

To deliver dsRNA to insects, dsRNA was complexed with an SPc nanocarrier and detergent (LIBY, Guangzhou, China) as described previously [29]. Both the dsRNA and the SPc nanocarrier were adjusted to a final concentration of 500 ng/μL. A 0.2 μL volume of the dsRNA/nanocarrier/detergent mixture or dsGFP/nanocarrier/detergent mixture (control) was topically applied to the abdominal tergum of 30 healthy fourth-instar wingless nymphs using a fine-tipped micropipette (Eppendorf, Hamburg, Germany). To assess silencing efficiency, 20 surviving aphids per treatment were collected at 24 and 48 h after treatment for qPCR analysis. Each treatment included three independent biological replicates.

To clarify the function of each gene, 90 healthy fourth-instar apterous nymphs in each treatment group were selected and treated with the dsRNA of the target gene or dsGFP. After 24 h of dsRNA treatment, the aphids from each group were randomly selected and injected with either an *E. coli* or *S. aureus* suspension. The groups without bacterial injection were used as the control groups. Mortality was recorded every 3 h after bacterial injection, and observation was continued until 48 h. After dsRNA treatment, the aphids were transferred onto fresh soybean leaves, which were spread flat on Petri dishes containing 2% (*w*/*v*) agar medium, for mortality monitoring. Survival curves were plotted based on the mortality recorded at each time point, and the differences in survival among the different treatment groups were compared.

### 2.7. Statistical Analysis

Statistical analyses were performed using GraphPad Prism 10.0 (GraphPad Software, San Diego, CA, USA). Differences in gene expression levels across developmental stages were analyzed by one-way ANOVA followed by Tukey’s post hoc test. For bacterial challenge and RNAi assays, relative expression levels were compared with those in the corresponding control groups using Student’s *t* test. Survival curves were analyzed using the log-rank (Mantel–Cox) test. Differences were considered significant at *p* < 0.05.

## 3. Results

To investigate the potential role of the JAK/STAT signaling pathway in the immune defense of *A. glycines*, we first identified the major pathway-associated genes and characterized their structural features. We then examined their developmental expression patterns, transcriptional responses to bacterial challenge, and functional roles in antibacterial defense through RNAi-based assays.

### 3.1. The JAK/STAT Signaling Pathway Components in A. glycines

**Figure 1:**
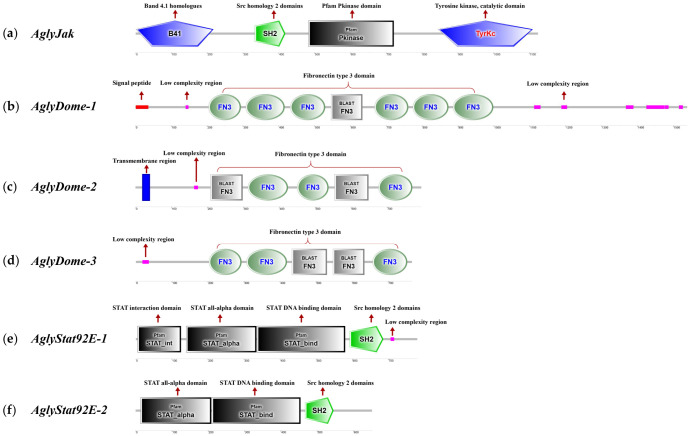
Schematic of the domain organization of putative JAK/STAT pathway proteins in *A. glycines*. (**a**) The predicted domain architecture includes a Band 4.1 domain (B41), a Src homology 2 domain (SH2), a tyrosine kinase catalytic domain (TyrKc), and a Pfam Pkinase domain (Pkinase). (**b**) The domain architecture comprises a signal peptide, fibronectin type III (FN3) domains, and multiple low-complexity regions. (**c**) The domain architecture comprises a transmembrane region, a low-complexity region, and FN3 domains. (**d**) The domain architecture comprises a low-complexity region and FN3 domains. (**e**) The domain architecture comprises a STAT interaction domain (STAT_int), a STAT all-alpha domain (STAT_alpha), a STAT DNA binding domain (STAT_bind), a Src homology 2 domain, and a low-complexity region. (**f**) The domain architecture comprises a STAT all-alpha domain, a STAT DNA binding domain, and Src homology 2 domains.

**Figure 2:**
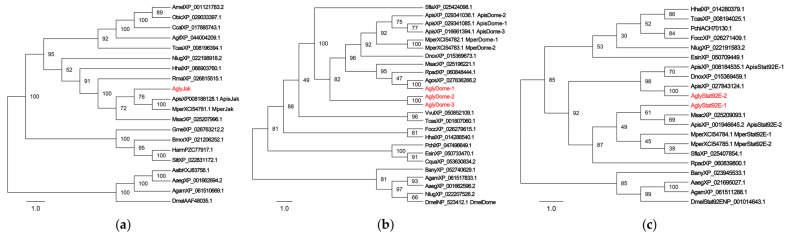
Phylogenetic analysis of JAK/STAT pathway protein sequences from *A. glycines* and other insect species. Red text highlights protein sequences from *A. glycines*. The phylogenetic trees represent AglyJak (**a**), AglyDome-1/2/3 (**b**), and AglyStat92E-1/2 (**c**). Aaeg, *Aedes aegypti*; Aalb, *Aedes albopictus*; Agam, *Anopheles gambiae*; Agif, *Aphidius gifuensis*; Agos, *Aphis gossypii*; Amel, *Apis mellifera*; Apis, *Acyrthosiphon pisum*; Bany, *Bicyclus anynana*; Bmor, *Bombyx mori*; Ccal, *Ceratina calcarata*; Cqua, *Cherax quadricarinatus*; Dmel, *D. melanogaster*; Dnox, *Diuraphis noxia*; Esin, *Eriocheir sinensis*; Focc, *Frankliniella occidentalis*; Gmel, *Galleria mellonella*; Harm, *Helicoverpa armigera*; Hhal, *Halyomorpha halys*; Mper, *Myzus persicae*; Msac, *Melanaphis sacchari*; Nlug, *Nilaparvata lugens*; Obic, *Osmia bicornis*; Pchi, *Penaeus chinensis*; Rmai, *Rhopalosiphum maidis*; Rpad, *Rhopalosiphum padi*; Sfla, *Sipha flava*; Slit, *Spodoptera litura*; Tcas, *Tribolium castaneum*; and Vvul, *Vespula vulgaris*.

To investigate the role of the JAK/STAT signaling pathway in the antibacterial defense of *A. glycines*, we first identified the core pathway-associated genes and characterized their structural features. Through homology searches, we identified six putative JAK/STAT pathway-associated genes in *A. glycines*: *AglyJak*, *AglyDome-1*, *AglyDome-2*, *AglyDome-3*, *AglyStat92E-1*, and *AglyStat92E-2*; the predicted proteins were 1124, 1539, 795, 766, 780, and 651 amino acids in length (GenBank accession numbers KAE9523455, KAE9525176, KAE9528782, KAE9528568, KAE9545698, and KAE9533143, respectively). The detailed physicochemical properties of these proteins are listed in Table 2. Conserved-domain analysis revealed that the AglyJak protein contains a 4.1 homologous domain (B41), an Src homology 2 domain (SH2), a tyrosine kinase catalytic domain (TyrKc), and a Pfam Pkinase domain (Pkinase) (Figure 1a). All three AglyDome proteins contain fibronectin type III domains and low-complexity regions; however, only AglyDome-1 contains a signal peptide, and AglyDome-2 contains a transmembrane region (Figure 1b–d). Both AglyStat92E isoforms contain a STAT all-alpha (STAT_alpha) domain, a STAT DNA-binding (STAT_binding) domain, and a Src homology 2 domain (SH2), whereas AglyStat92E-1 additionally contains a STAT interaction domain (STAT_int) and a low-complexity region (Figure 1e–f). Phylogenetic analysis revealed that the six *A. glycines* JAK/STAT proteins each clustered into well-supported clades with orthologs from related hemipteran aphids, supporting their evolutionary conservation within Hemiptera/Aphididae (Figure 2). These results identified six conserved JAK/STAT pathway-related genes in *A. glycines* and confirmed that their predicted protein structures are consistent with known pathway components.

### 3.2. Developmental Expression of JAK/STAT Genes

**Figure 3:**
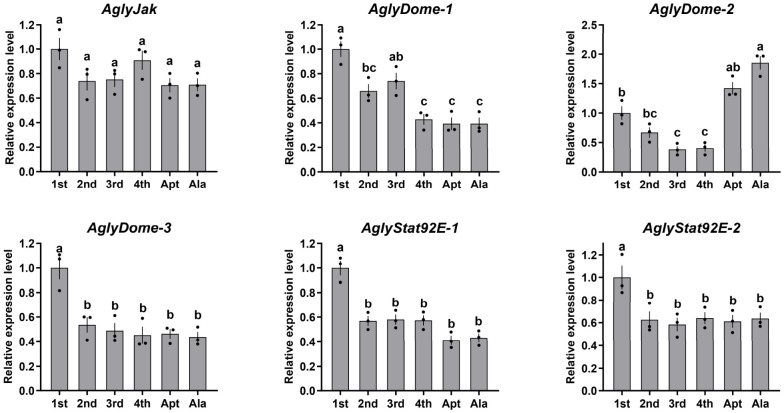
Expression levels of key JAK/STAT pathway genes in *A. glycines* across developmental stages. The 1st, 2nd, 3rd, and 4th represent first-, second-, third-, and fourth-instar nymphs, respectively; Apt and Ala represent apterous and alate adults, respectively. The data are presented as the mean ± SEM (*n* = 3). Bars sharing the same letter are not significantly different (one-way ANOVA, Tukey’s HSD post hoc test; *p* < 0.05).

After identifying the major JAK/STAT pathway components in *A. glycines*, we next examined their expression patterns across developmental stages. The developmental expression profiles of six JAK/STAT pathway genes in *A. glycines* are shown in Figure 3. The expression of *AglyJak* changed little throughout development, with no significant differences observed. In contrast, *AglyDome-1* transcripts were most abundant in first-instar nymphs and then gradually decreased during development, reaching their lowest levels in fourth-instar nymphs and adults. The expression level of *AglyDome-2* was the lowest in third- and fourth-instar nymphs but significantly increased in adults, with the highest transcript abundance observed in alate adults. In addition, *AglyDome-3*, *AglyStat92E-1*, and *AglyStat92E-2* displayed similar expression patterns. All three genes were expressed at their highest levels in first-instar nymphs, after which transcript abundance decreased sharply from the second instar onward and remained low throughout later nymphal stages and adulthood. Overall, most JAK/STAT pathway genes exhibited stage-specific expression patterns, with several showing relatively high transcript abundance in early nymphal stages.

### 3.3. Bacteria-Induced Expression of JAK/STAT Genes

**Figure 4:**
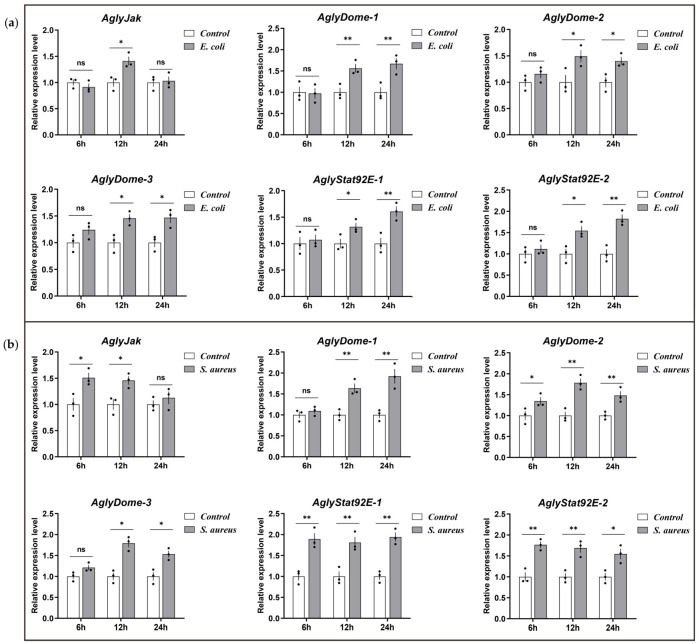
Expression of key JAK/STAT pathway genes in *A. glycines* following challenge with *E. coli* and *S. aureus*. (**a**) Relative expression levels of *AglyJak*, *AglyDome-1/2/3*, and *AglyStat92E-1/2* after infection with *E. coli*. (**b**) Relative expression levels of *AglyJak*, *AglyDome-1/2/3*, and *AglyStat92E-1/2* after infection with *S. aureus*. The data are presented as the mean ± SEM (*n* = 3). Asterisks denote significant differences (Student’s *t* test, * *p* < 0.05, ** *p* < 0.01, ns, not significant). Control: Sterile 0.85% (*w*/*v*) NaCl solution, matching the vehicle used to suspend *E. coli* and *S. aureus* inocula.

Following the analysis of developmental expression patterns, we next investigated whether these JAK/STAT pathway genes respond to bacterial challenge in *A. glycines*. The transcriptional responses of six JAK/STAT pathway genes in *A. glycines* to bacterial challenge were examined (Figure 4). These genes exhibited distinct temporal expression patterns depending on the bacterial strain. Following *E. coli* infection, no significant changes in transcript levels were detected at 6 h postinjection for any of the genes tested (Figure 4a). At 12 h, the *AglyJak* transcript levels were significantly upregulated, but the transcript level returned to control levels by 24 h (Figure 4a). In contrast, *AglyDome-1*, *AglyDome-2*, *AglyDome-3*, *AglyStat92E-1*, and *AglyStat92E-2* were significantly upregulated at both 12 h and 24 h (Figure 4a). Following *S. aureus* infection, *AglyJak* expression increased significantly at 6 h and 12 h but not at 24 h (Figure 4b). Although *AglyDome-1* and *AglyDome-3* showed no significant change at 6 h, both genes were significantly upregulated at 12 and 24 h (Figure 4b). In contrast, *AglyDome-2*, *AglyStat92E-1*, and *AglyStat92E-2* were significantly induced as early as 6 h, and this upregulation persisted until 24 h (Figure 4b). Overall, these results indicate that multiple JAK/STAT pathway genes in *A. glycines* are transcriptionally activated by bacterial infection, supporting their potential involvement in antibacterial immune responses.

### 3.4. RNAi Silencing of JAK/STAT Genes Increases Bacterial Susceptibility

**Figure 5:**
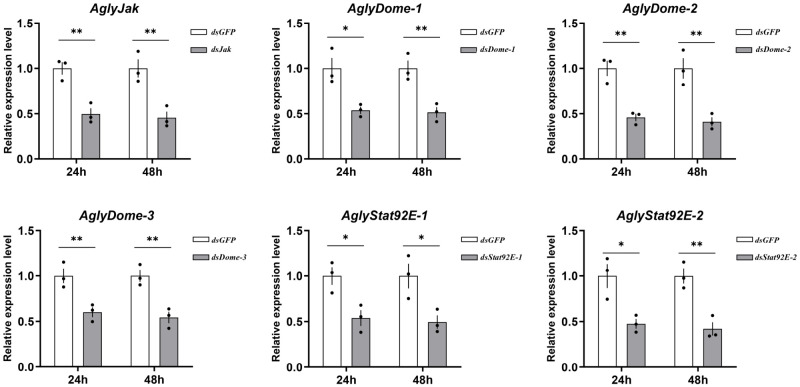
Expression of key JAK/STAT pathway genes in *A. glycines* after dsRNA-mediated knockdown. The data are presented as the mean ± SEM (*n* = 3). Asterisks indicate significant differences relative to the control (Student’s *t* test; * *p* < 0.05, ** *p* < 0.01).

**Figure 6:**
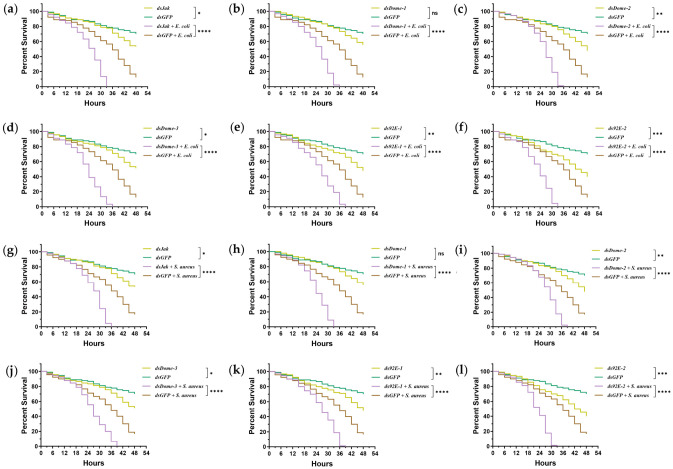
Effects of JAK/STAT gene knockdown on *A. glycines* survival after bacterial challenge. The dsGFP + bacterial challenge group was used as the infected RNAi control group for comparison with target-gene dsRNA-treated aphids under the same bacterial challenge. (**a**–**f**) Survival of *A. glycines* following *E. coli* infection after the knockdown of *AglyJak* (**a**), *AglyDome-1/2/3* (**b**–**d**), and *AglyStat92E-1/2* (**e**–**f**). (**g**–**l**) Survival following *S. aureus* infection after the knockdown of the same set of genes. Survival curves were compared using the log-rank (Mantel–Cox) test (*n* = 90; ns, not significant; * *p* < 0.05, ** *p* < 0.01, *** *p* < 0.001, **** *p* < 0.0001).

To further determine whether the bacteria-induced JAK/STAT pathway genes contribute to antibacterial defense in *A. glycines*, we performed RNAi-based functional analyses. The efficiency of dsRNA-mediated knockdown of the six JAK/STAT pathway genes in *A. glycines* was assessed by qPCR (Figure 5). Transcript levels of all target genes were significantly reduced at both 24 and 48 h after treatment relative to the dsGFP control. Specifically, compared with the dsGFP control group, transcript levels were reduced at both time points as follows: *AglyJak* to 49.7% and 45.6%, *AglyDome-1* to 53.7% and 51.5%, *AglyDome-2* to 45.8% and 41.1%, *AglyDome-3* to 60.1% and 54.4%, *AglyStat92E-1* to 53.9% and 49.5%, and *AglyStat92E-2* to 47.6% and 42.1%, respectively. After these genes were knocked down, the soybean aphids were challenged with *E. coli* or *S. aureus*. The results revealed that silencing any of the tested JAK/STAT pathway genes significantly increased the mortality rates of the aphids following bacterial infection (Figure 6). Specifically, at 24 h after *E. coli* injection, the mortality rates in *AglyJak*, *AglyDome-1*, *AglyDome-2*, *AglyDome-3*, *AglyStat92E-1*, and *AglyStat92E-2* RNAi groups were 48.9%, 46.7%, 40.0%, 61.1%, 44.4%, and 58.9%, respectively, compared with 26.7% in the dsGFP + *E. coli* control group. Similarly, at 24 h after *S. aureus* injection, the mortality rates in the corresponding RNAi-silenced groups were 41.1%, 55.6%, 32.2%, 44.4%, 41.1%, and 58.9%, respectively, compared with 28.9% in the dsGFP + *S. aureus* control group. These findings demonstrate that JAK/STAT pathway genes contribute substantially to the antibacterial defense of *A. glycines*, as their silencing markedly increases aphid susceptibility to bacterial infection.

## 4. Discussion

Consistent with the objectives of this study, we first describe the identification and structural characteristics of the JAK/STAT pathway components in *A. glycines* as a basis for subsequent functional interpretation. The JAK/STAT signaling pathway is a widely conserved immune regulatory pathway in insects [12,14]. Although its importance in insect immunity has been demonstrated in various species [19,20,34], its function in the soybean aphid has not been experimentally validated. In this study, we identified and characterized six genes associated with the JAK/STAT pathway in *A. glycines*: *AglyJak*, *AglyDome-1*, *AglyDome-2*, *AglyDome-3*, *AglyStat92E-1*, and *AglyStat92E-2*. Conserved-domain analysis revealed that AglyJak contains the canonical B41, SH2, protein kinase (Pkinase), and TyrKc domains. All three *AglyDome* homologous genes encode proteins that contain multiple FN3 domains. In AglyStat92E, both AglyStat92E-1 and AglyStat92E-2 contain STAT_alpha, STAT_bind, and SH2 domains. AglyStat92E-1 additionally contains a STAT_int domain that is absent in AglyStat92E-2. These structural features are consistent with those of homologous proteins reported in *D. melanogaster* and *M. persicae* [14,25]. However, the Dome-like proteins differ in domain architecture, suggesting possible structural divergence or functional differentiation. Phylogenetic analysis further supported the identity of these proteins, as each clustered with homologous proteins from other aphid species. Overall, these results support the presence of conserved JAK/STAT pathway components in *A. glycines*.

Building on the identification of these pathway components, we next consider what their developmental expression patterns may suggest about their biological roles in the soybean aphid. Gene expression analysis across developmental stages revealed that first-instar nymphs exhibited the highest transcript levels of *AglyDome-1*, *AglyDome-3*, *AglyStat92E-1*, and *AglyStat92E-2*. In insects, the stage-specific differential expression of immune genes is commonly observed. For example, higher expression levels of *MperJak* and *MperStat92E-2* have been reported in first-instar nymphs of *M. persicae* [25]. Similarly, in *D. melanogaster*, the expression of antimicrobial peptides and the immune response significantly differ among different developmental stages [11,35]. Ecologically, insects are often more vulnerable during early developmental stages and must allocate limited energy resources to rapid growth [36]. An early study indicated that the survival and development of early-stage soybean aphids are critical for subsequent population growth [37]. Therefore, the elevated expression of JAK/STAT pathway genes in first-instar nymphs may reflect stage-specific immune investment during an ecologically vulnerable developmental period, although this hypothesis requires further functional validation. Overall, these developmental data suggest that several JAK/STAT pathway genes may play particularly important roles during the early nymphal stage of *A. glycines*.

In addition to developmental regulation, the bacterial induction results provide further insight into whether these genes are directly involved in immune responses to infection. The present study shows that bacterial infection significantly increased the transcriptional levels of *AglyJak*, *AglyDome*, and *AglyStat* in *A. glycines*, suggesting that these JAK/STAT-associated genes are responsive to infection in *A. glycines*. These findings are inconsistent with previous studies in other insects. For example, in *Drosophila*, septic injury can induce the expression of the hemocyte-specific *upd3* gene, which is required for activation of JAK/STAT-dependent target genes in the fat body, and these results are consistent with the view that bacterial infection can induce JAK/STAT-related transcriptional responses in insects [38]. In addition, in *A. pisum* and *M. persicae*, bacterial infection also induces transcription of JAK/STAT signaling pathway genes [24,25]. Compared with the sustained induction of *AglyDome* and *AglyStat*, *AglyJak* exhibited a more transient transcriptional response. These two differential expression patterns suggest that the continuous expression of *Dome* and *Stat* may support maintenance of the immune response, whereas *Jak* may function primarily during the early phase of the immune activation, when rapid signaling is required. This temporal regulation is consistent with the current understanding of insect JAK/STAT signaling [12,14]. Thus, the transcriptional evidence indicates that JAK/STAT pathway-related genes in *A. glycines* are infection-responsive and may contribute to different phases of the antibacterial immune response.

To further assess whether these transcriptional responses are functionally meaningful, RNAi experiments were performed to directly evaluate the contribution of this pathway to antibacterial defense. In addition to the transcriptional response, the RNAi results further indicated that the JAK/STAT pathway is important for the antibacterial defense in the soybean aphid. Previous studies in various insects have also demonstrated the role of this pathway in host resistance to infection. For example, knocking down STAT with RNAi increased the number of *Plasmodium vivax* oocysts in the midgut of *Anopheles aquasalis* and increased the susceptibility of *A. gambiae* to *P. berghei* and *P. falciparum* [39,40]. Similar findings were observed in sand flies, in which STAT knockdown suppressed the expression of inducible nitric oxide synthase and promoted the proliferation of *Leishmania* [41]. Recent studies in aphids have shown that silencing JAK/STAT components can significantly increase the mortality rate after bacterial infection [24,25]. Our findings align with these precedents: suppressing *AglyJak*, *AglyDome*, or *AglyStat* markedly reduced soybean aphid survival following challenge with *E. coli* or *S. aureus*. Although these results suggest that the JAK/STAT pathway contributes substantially to antibacterial defense in *A. glycines*, the underlying physiological mechanisms, such as direct bacterial clearance or changes in host tolerance, still require further investigation. Therefore, the RNAi results provide functional evidence that the JAK/STAT pathway is an important component of the antibacterial immune defense in *A. glycines*.

Given their functional importance, these findings may also have potential implications for the development of alternative strategies for soybean aphid management. Chemical insecticides remain the primary means of controlling soybean aphids. However, excessive reliance on chemical control may accelerate the evolution of insecticide resistance and compromise long-term control efficiency. The resistance of *A. glycines* to major classes of insecticides, including neonicotinoids and pyrethroids, has already been documented [42,43], highlighting the need for alternative and more sustainable management strategies. The results of this study suggest that JAK/STAT pathway genes may represent potential molecular targets for the control of *A. glycines*. As silencing *AglyJak*, *AglyDome*, or *AglyStat* reduced aphid survival following bacterial challenge, disruption of this pathway may impair host immune defense and thereby increase soybean aphid susceptibility to microbial control agents. RNA interference (RNAi) provides a potential means of exploiting these targets for species-selective pest management [28]. Recent advances in nanomaterial-based dsRNA delivery systems have improved dsRNA stability and delivery efficiency, thereby enhancing RNAi efficacy [29,44]. Moreover, previous studies in aphids and other insect pests suggest that suppression of host immune defenses can enhance susceptibility to entomopathogenic microorganisms, including *Beauveria bassiana* [45,46]. Collectively, these findings suggest that JAK/STAT pathway genes may serve as promising candidate targets for RNAi-assisted or microbially based management of *A. glycines*.

Despite the clear patterns observed in this study, several limitations should be considered when interpreting the results. Although bacterial challenge induced the expression of *AglyJak*, *AglyDome*, and *AglyStat*, and RNAi-mediated silencing increased aphid mortality, the underlying mechanisms were not directly investigated. It therefore remains unclear whether the reduced survival resulted from impaired bacterial clearance, disruption of immune signaling, decreased host tolerance, or a combination of these effects. This question is particularly relevant in aphids, whose immune systems differ from those of many other insects in both gene repertoire and regulatory organization [23]. Nevertheless, the available evidence supports the principal conclusion of this study. Conserved JAK/STAT-related genes were identified in *A. glycines*, and all tested genes were induced by both *E. coli* and *S. aureus*. Moreover, silencing each gene consistently increased aphid susceptibility to bacterial infection. Similar findings in other aphid species provide additional support for this interpretation [24,25]. Future studies should quantify bacterial loads, identify downstream immune effectors, characterize tissue-specific responses, and evaluate improved dsRNA delivery strategies under more biologically relevant conditions.

## 5. Conclusions

Overall, the results of this study provide functional evidence that JAK/STAT pathway-related genes contribute to antibacterial defense in the soybean aphid and advance our understanding of immune regulation in this species. Functional analyses of *AglyJak*, *AglyDome*, and *AglyStat* further suggest that these genes may represent candidate molecular targets for the future management of *A. glycines*. Because gene silencing reduced aphid survival following bacterial infection, disruption of the JAK/STAT pathway may weaken host immune defenses and potentially enhance susceptibility to microbial control agents. Supporting this possibility, RNAi-mediated silencing of *ApGNBP1* in *A. pisum* has been shown to suppress phenoloxidase activity and increase aphid susceptibility to *B. bassiana* infection [45]. In summary, our findings establish JAK/STAT pathway-related genes as functionally important components of antibacterial defense in the soybean aphid and identify them as potential targets for future RNAi-assisted or microbially based pest management strategies.

## Figures and Tables

**Table 1:** Sequences of primers used for qRT-PCR and dsRNA synthesis.

	Gene	Forward Primer Sequence (5′–3′)	Reverse Primer Sequence (5′–3′)	Size (bp)
Quantitative PCR	*AglyJak*	TGACACATGCAAACGAGAGC	TGCTTAACTACTTCTTGGGGCA	132
	*AglyDome-1*	ATGGGAATGACACCGCTGAC	AGCCGCTGTTGAGTTGATGA	119
	*AglyDome-2*	ACGCATGTCCCTGGTTCAG	TTCCCACACCATTCCGCTAC	165
	*AglyDome-3*	TCCTCCAGCAAGTTTCGATGT	GCACACGCCGATGTCTGAA	178
	*AglyStat92E-1*	TGTCTCCTATTACGGCTTGGGA	TGGGGATAACAACTGCCTCAC	110
	*AglyStat92E-2*	GGACAGACGGAGCTATAATGGG	GCAACAGTTAGTCCACCCAATT	124
	*β-Tub* ^1^	GTCAGTGCGGAAACCAGATC	TGGCACGGGGTACATACTTT	162
	*EF1α* ^1^	GGCTGATTGTGCTGTGCTTA	TCGCTGTATGGTGGTTCAGT	168
	*RPS12* ^1^	CCCAAGTTAACGGCAGTCTT	CCCAAGTTAACGGCAGTCTT	196
dsRNA synthesis	*AglyJak*	**T7-**TCTCATGGACCAATCAGCA ^2^	**T7-**AGCACTCGAAGACACAGA	597
	*AglyDome-1*	**T7-**GCGTTCGCTTCCCATAAAAGT	**T7-**GGCAACACACAACTGACAGAC	435
	*AglyDome-2*	**T7-**TCCAAGCCACAGGATCGC	**T7-**ATTCAAACACACGCCAAGTAGT	310
	*AglyDome-3*	**T7-**ACATGCATTTTAAATCCTGATCACG	**T7-**TGGTATCCAATCGTGTTAATGCT	320
	*AglyStat92E-1*	**T7-**TTCCTATTGAAGTGAGGCAGTT	**T7-**TTTACATTGACTTCCATCAGAGAA	319
	*AglyStat92E-2*	**T7-**AGCAGAAGTACGCCTATTGA	**T7-**AGCTGCGGAAAATTTCATGT	505
	*GFP*	**T7-**AAGAGTGCCATGCCCGAAG	**T7-**TGTGTAATCCCAGCAGCAGT	440

^1^ Primers for internal reference genes were as reported previously [32]. ^2^ The T7 promoter sequence (TAATACGACTCACTATAGGG) is indicated by “**T7-**“.

**Table 2:** Bioinformatic characteristics of JAK/STAT pathway-associated genes in *Aphis glycines*.

Gene	Accession No.	ORF (Amino Acids)	Molecular Weight (kDa)	Isoelectric Points	Leucine Ratio (%)	Instability Index
*AglyJak*	KAE9523455	1124	129.18	6.05	10.3	46.04
*AglyDome-1*	KAE9525176	1539	171.18	6.37	6.0	50.14
*AglyDome-* *2*	KAE9528782	795	90.49	6.27	7.2	41.33
*AglyDome-* *3*	KAE9528568	766	86.93	6.15	7.0	32.04
*AglyStat92E-1*	KAE9545698	780	89.32	5.97	9.4	39.60
*AglyStat92E-* *2*	KAE9533143	651	74.75	8.23	9.4	37.79

## Data Availability

The original contributions presented in this study are included in the article. Further inquiries can be directed to the corresponding author.

## Supplementary Materials

The following supporting information can be downloaded at: https://www.mdpi.com/article/10.3390/insects17070687/s1; Table S1: Best-fit substitution models for phylogenetic analysis.
