# Supplementary material for: The JAK/STAT Signaling Pathway Mediates Antibacterial Immunity in the Soybean Aphid Aphis glycines
Source: Insects. 2026 Jul 1;17(7):687. doi: 10.3390/insects17070687 (PMC13409945; doi:10.3390/insects17070687)
Supplement: Supplementary file 1 [file insects-17-00687-s001.zip › insects-4387390-supplementary.pdf]

Table S1. Best-fit substitution models for phylogenetic analysis.

Protein	Best-fit substitution models
Jak	Q.insect+F+I+G4
Dome	VT+F+R4
Stat92E	Q.insect+F+I+R3
